# Patient-Relevant Digital-Motor Outcomes for Clinical Trials in Hereditary Spastic Paraplegia Type 7: A Multicenter PROSPAX Study

**DOI:** 10.1212/WNL.0000000000209887

**Published:** 2024-12-02

**Authors:** Lukas Beichert, Jens Seemann, Christoph Kessler, Andreas Traschütz, Doreen Müller, Katrin Dillmann-Jehn, Ivana Ricca, Sara Satolli, Nazli A. Basak, Giulia Coarelli, Dagmar Timmann, Cynthia Gagnon, Bart P.C. van de Warrenburg, for the PROSPAX Consortium, Winfried Ilg, Matthis Synofzik, Rebecca Schüle

**Affiliations:** Division Translational Genomics of Neurodegenerative Diseases (L.B., A.T., D.M., M.S.), Hertie-Institute for Clinical Brain Research and Center for Neurology, and German Center for Neurodegenerative Diseases (DZNE) (L.B., A.T., D.M., K.D.-J., M.S., R.S.), University of Tübingen; Section Computational Sensomotorics (J.S., W.I.), Hertie Institute for Clinical Brain Research; Centre for Integrative Neuroscience (CIN) (J.S., W.I.); Department of Neurodegenerative Diseases (C.K.), Hertie-Institute for Clinical Brain Research and Center for Neurology, University of Tübingen; Center for Neurology and Hertie Institute for Clinical Brain Research (K.D.-J., R.S.), University Hospital Tübingen, Germany; Molecular Medicine (I.R., S.S.), IRCCS Fondazione Stella Maris, Pisa, Italy; Koç University (N.A.B.), Translational Medicine Research Center, KUTTAM-NDAL, Istanbul, Turkey; Sorbonne Université (G.C.), Paris Brain Institute, INSERM, CNRS, APHP, France; Department of Neurology and Center for Translational Neuro- and Behavioral Sciences (D.T.), University Hospital Essen, University of Duisburg-Essen, Germany; Groupe de recherche interdisciplinaire sur les maladies neuromusculaires (GRIMN) (C.G.), Centre intégré universitaire de santé et de services sociaux du Saguenay-Lac-St-Jean; Centre de recherche du Centre intégré universitaire de santé et de services sociaux du Saguenay-Lac-St-Jean (C.G.); Faculté de médecine et des sciences de la santé (C.G.), Université de Sherbrooke, Québec, Canada; Department of Neurology (B.P.C.v.d.W.), Radboud University Medical Center, Nijmegen, the Netherlands; and Division of Neurodegenerative Diseases (R.S.), Department of Neurology, Heidelberg University Hospital, Germany.

## Abstract

**Background and Objectives:**

With targeted treatment trials on the horizon, identification of sensitive and valid outcome measures becomes a priority for >100 spastic ataxias. While digital-motor measures, assessed using wearable sensors, are considered prime outcome candidates for spastic ataxias, genotype-specific validation studies are lacking. We here aimed to identify candidate digital-motor outcomes for spastic paraplegia type 7 (SPG7)—one of the most common spastic ataxias—that (1) reflect patient-relevant health aspects, even in mild, trial-relevant disease stages; (2) are suitable for a multicenter setting; and (3) assess mobility also during uninstructed walking simulating real life.

**Methods:**

This cross-sectional multicenter study (7 centers, 6 countries) analyzed defined laboratory-based walking and uninstructed “supervised free walking” in patients with SPG7 and healthy controls using 3 wearable sensors (Opal APDM). For the extracted digital gait measures, we assessed effect sizes for the discrimination of patients and controls (Cliff δ) and Spearman correlations with measures of functional mobility and overall disease severity (Spastic Paraplegia Rating Scale [SPRS], including mobility subscore SPRS^mobility^; Scale for the Assessment and Rating of Ataxia [SARA]) and the activities of daily living subscore of the Friedreich Ataxia Rating Scale (FARS-ADL).

**Results:**

Gait was analyzed in 65 patients with SPG7 and 50 healthy controls. Among 30 hypothesis-based gait measures, 18 demonstrated at least moderate effect size (δ > 0.5) in discriminating patients from controls and 17 even in mild disease stages (SPRS^mobility^ ≤ 9, n = 41). Spatiotemporal variability measures such as spatial variability measure SPcmp (ρ = 0.67, *p* < 0.0001) and stride time CV (ρ = 0.67, *p* < 0.0001) showed the largest correlations with functional mobility (SPRS^mobility^)—as with overall disease severity (SPRS, SARA) and activities of daily living (FARS-ADL). The correlations of variability measures with SPRS^mobility^ could be confirmed in mild disease stages (e.g., SPcmp: ρ = 0.50, *p* < 0.0001) and in “supervised free walking” (e.g., stride time CV: ρ = −0.57, *p* < 0.0001).

**Discussion:**

We here identified trial-ready digital-motor candidate outcomes for the spastic ataxia SPG7 with proven multicenter applicability, ability to discriminate patients from controls, and correlation with measures of patient-relevant health aspects—even in mild disease stages. If validated longitudinally, these sensor outcomes might inform future natural history and treatment trials in SPG7 and other spastic ataxias.

## Introduction

As we are entering the era of genomic therapies for rare diseases, the identification of reliable and valid outcome parameters with sensitivity to longitudinal change and treatment effects is becoming a priority task. Quantitative digital-motor outcomes, assessed through body-worn sensors, could potentially meet this need by capturing changes in patient-relevant health aspects within trial-like time frames, even in moderately progressive diseases such as hereditary spastic paraplegia type 7 (SPG7).^1^

SPG7 is an autosomal recessive hereditary neurodegenerative disease, manifesting as spastic ataxia typically in adulthood.^2^ Although currently no curative treatment exists, progress in the development of genomic therapies for genetic spastic ataxias, including antisense oligonucleotides and gene replacement strategies, and first treatment trials conducted in diseases such as spastic paraplegia type 50 (SPG50, NCT05518188) or spinocerebellar ataxia types 1 and 3 (SCA1/SCA3, NCT05822908) raises hopes that potential therapeutic agents for SPG7 might become available in the near future.

Digital gait measures have demonstrated promising properties in hereditary ataxias, reliably discriminating between patients and controls, correlating cross-sectionally with clinical measures of disease severity, and—in the few longitudinal studies conducted so far—also exhibiting sensitivity to capturing change.^3-7^ By contrast, only few studies on sensor-based gait analysis have been conducted in hereditary spastic paraplegias (HSPs),^8,9^ and no study has yet systematically used body-worn sensors to examine gait in diseases such as SPG7 or other spastic ataxias. Anticipated treatment trials in diseases such as SPG7 will likely (1) focus on patients in early disease stages and (2) involve multiple centers to reach sufficient statistical power to detect small changes over time. Validation of sensor gait measures as digital-motor performance outcomes for treatment trials^10^ should, therefore, specifically assess performance in those early disease severity strata and demonstrate multicenter applicability. Moreover, for sensor gait measures to gain regulatory and patient acceptance as outcome parameters, they need to reflect health aspects that are relevant to patients.^10^ Therefore, performance of sensor outcomes needs to be assessed in patient-relevant settings, that is, conditions resembling patients' everyday lives, and gait measures should demonstrate correlation with clinical measures capturing disease-related impairment on a functional, patient-relevant level.

This cross-sectional study presents candidate digital gait outcomes for spastic ataxias such as SPG7, demonstrating discriminative power and correlation with clinical outcome assessments of patient-relevant health aspects. Evaluated across multiple centers, in gait assessments in settings simulating real life, and with a special focus on patients in mild disease stages, the presented gait measures are prime candidate outcomes to be validated longitudinally and potentially be applied in future treatment trials.

## Methods

### Standard Protocol Approvals, Registrations, and Patient Consents

The Institutional Review Board of the University of Tübingen approved the study (AZ 824/2019BO2). All participants provided written informed consent before participation according to the Declaration of Helsinki. The study was registered as observational (patient registry) study at ClinicalTrials.gov (No.: NCT04297891).

### Participants

The study cohort was part of the study “An integrated multimodal progression chart in spastic ataxias” (PROSPAX) funded by the European Union through the European Joint Programme on Rare Diseases. 70 patients with genetically confirmed and clinically manifest SPG7 (defined as presence of biallelic likely pathogenic or pathogenic SPG7 variants according to American College of Medical Genetics and Genomics criteria) and 50 healthy controls (HCs) with available gait recordings were recruited by 7 centers in 6 countries based on the following inclusion criteria: (1) ability to walk at least 10 m without walking aid; (2) absence of severe comorbidities (due to SPG7 or unrelated) that present a major confounder for evaluation of gait and stance such as amputation, blindness, severe dementia, severe joint deformities, or contractures significantly interfering with gait analysis (as assessed by clinical judgment) or fixed orthoses. HCs had no history of any neurologic or psychiatric disease and no family history of neurodegenerative disease and did not show any neurologic signs on clinical examination. After exclusion of invalid gait recordings (damaged data files, unreliable step detection), recordings of 65 patients with SPG7 and 50 HCs from the laboratory-based walking (LBW) condition and of 57 patients and 37 HCs from the supervised free walking (SFW) condition remained suitable for analysis (eFigure 1).

### Clinical Assessments

All participants underwent a detailed neurologic examination. Disease severity was rated using the Scale for the Assessment and Rating of Ataxia (SARA)^11^ and the Spastic Paraplegia Rating Scale (SPRS).^12^ Mobility-relevant SPRS items 1–6 were combined into a subscore termed SPRS^mobility^.^13^ SARA items 1–3 rating gait and posture were combined into the SARA posture and gait subscore (SARA^PG^).^3,14^ The activities of daily living subscore of the Friedreich Ataxia Rating Scale (FARS-ADL) was used to assess impact of the disease on patient-relevant health aspects, and the Friedreich Ataxia Rating Scale Functional Staging (FARS staging) was used to classify patients by functional disease stages.^15^

### Gait Conditions

Walking movements of patients wearing comfortable shoes (e.g., sports shoes)^1^ were recorded under 2 different conditions:

1. LBW condition: walking was constrained by a specified walking distance of 10 m in a specific quiet nonpublic indoor floor and supervised by a study assessor watching the walking performance; participants were instructed to walk back and forth the walking distance at a self-selected speed; participants were asked to halt after 1 minute of walking, and recordings were terminated.
2. SFW condition: largely unconstrained walking was measured in public spaces in an institutional (hospital) compound (all indoor: 4 study sites; indoor and outdoor: 3 study sites), where participants were free to choose the walking speed as they were led along a predefined route lasting approximately 5–10 minutes; participants were accompanied by a study assessor watching their walking performance.

### Gait Measures

3 inertial sensors (Opal, APDM Wearable Technologies Inc., Portland, WA) were attached on both feet and the posterior trunk at the level of L5 with elastic Velcro bands. Inertial sensor data were collected and wirelessly streamed to a laptop for automatic generation of gait and balance metrics using Mobility Lab software (APDM, Inc.). For the SFW condition, data were logged on board of each Opal sensor and downloaded after the session. Step events and spatiotemporal gait features for each stride were extracted from the inertial measurement unit sensors using APDM's *Mobility Lab* software (version 2),^16^ which has been shown to deliver good-to-excellent accuracy and repeatability.^17,18^ For the LBW condition, only recordings with a minimum number of 20 detected strides were included in the analysis. For the SFW condition, only recordings from walking bouts of at least 5 consecutive strides were analyzed.

We considered a hypothesis-based selection of 30 candidate gait measures based on previous studies and literature (from both the ataxia and HSP fields) and clinical plausibility,^3,6,7,9,19-21^ and all 30 measures were evaluated in LBW and SFW. Gait measures for each recording were obtained through 3 approaches: (1) by computation of 1 or more of the following nonparametric measures for each of the 14 of the gait features extracted for each stride by *Mobility Lab*: median, normalized median absolute deviation (MADN = MAD/0.6745), and coefficient of variation (CV = median/MADN). For variability measures, we used CV when we hypothesized that variation of the measure would increase proportional to the median of the measure and MADN otherwise; (2) by computation of 1 composite measure of spatial step variability (SPcmp); (3) by computation of median harmonic ratio (HR) of raw accelerometer signals from the lumbar sensor in 3 directions. A list of all gait measures and their definitions is provided in eTable 1.

The composite measure SPcmp was formed from stride length CV and lateral step deviation to capture spatial step variability in both anterior-posterior and mediolateral directions in 1 measure.^3^ In brief, the composite measure was determined for each individual participant in 2 steps: In step 1, the relative value of the participant in comparison with the value range of all participants was calculated for each of the 2 constituent measures separately (resulting in values between 0 and 1). In step 2, the greater of these 2 relative values was selected.

HR of pelvis linear acceleration was determined to quantify the smoothness of motion, as described previously.^3^ In brief, this method quantifies the harmonic content of the acceleration signals in each direction (HR anterior-posterior [AP], mediolateral [ML], vertical [V]) using stride frequency as the fundamental frequency component. Using a finite Fourier series, HR is calculated by dividing the sum of the amplitudes of the first 10 even harmonics by the sum of the amplitudes of the first 10 odd harmonics for each given stride.^22,23^ A greater HR was interpreted as greater walking smoothness. HR measures have been shown to distinguish between patients with cerebellar disease and HCs under LBW and real-life walking conditions.^3,24^

### Statistics

For the hypothesis-based selection of 30 gait measures, the ability to discriminate between patients and controls was assessed—in LBW and SFW separately—by calculation of the nonparametric effect size measure Cliff δ.^25^ Discriminative effect sizes were classified as *small* (δ ≥ 0.3), *moderate* (δ ≥ 0.5), or *large* (δ ≥ 0.8). In addition, we performed a receiver operating characteristic (ROC) analysis and reported the area under the curve (AUC). Significance of group differences was determined by the nonparametric Wilcoxon signed-rank test. Group differences were considered significant when *p* < 0.05/n (n = 30: number of gait measures), accounting for multiple comparisons.

For gait measures that discriminated patients from controls with at least moderate effect sizes, we assessed convergent validity by examining Spearman correlation between gait measures and 5 clinical outcome measures. The scales SPRS^mobility^ as a measure of mobility and SARA^PG^ as a measure of posture and gait were considered the most direct clinical equivalents to the sensor-based gait measures while at the same time reflecting health aspects of high relevance to patients and were thus treated as primary outcomes. In addition, SPRS and SARA as standard measures of general disease severity in HSP and ataxia, respectively, and FARS-ADL as a measure of activities of daily living were included in the analysis as exploratory outcomes (because they measure aspects going beyond the construct measured by gait measures, such as speech, upper limb dexterity, and pain). Effect sizes ρ are displayed with 95% confidence intervals (determined by bootstrapping using MATLAB's *bootci* function with 2,000 samples) and *p* values. Effect sizes ρ were classified as *small* (ρ ≥ 0.1), *medium* (ρ ≥ 0.3), *large* (ρ ≥ 0.5), or very large (ρ ≥ 0.7).^26^ Correlations between gait measures and primary clinical outcomes were reported as significant when *p* < 0.05/(n × m) (n: number of discriminative gait measures, m = 2: number of primary clinical outcome measures), hereby correcting for the number of comparisons. Correlations with exploratory outcomes SPRS, SARA, and FARS-ADL were deemed as significant when *p* < 0.05. Furthermore, Spearman correlations between the 2 walking conditions (LBW, SFW) were calculated for each gait measure.

To evaluate the ability of the gait measures to discriminate mildly affected patients from controls, we performed a median split of the patient cohort with respect to SPRS^mobility^, thus defining a subgroup with mild (SPRS^mobility^ ≤ median_LBW_; termed “mild patient cohort”) and intermediate (SPRS^mobility^ > median_LBW_) disease severity, where median_LBW_ denotes the median SPRS^mobility^ of all patients with valid recordings of LBW.

To assess whether gait measures differentiated between disease stages defined by the FARS staging, thus determining approximate meaningful score regions, patients were grouped into 3 stages (mild: ≤2.0, intermediate: 2.5–3.5, advanced: ≥4.0). Significance of between-group/stage differences was analyzed using the Kruskal-Wallis test, and when the Kruskal-Wallis test yielded a significant effect, the Wilcoxon rank-sum test post hoc was used (considered significant when *p* < 0.05). Discrimination between groups/stages was further assessed by Cliff δ and ROC analysis (AUC).

Statistical analysis was performed using MATLAB (version R2022a).

### Data Availability

Data will be made available on reasonable request and as patient consent allows.

## Results

Gait recordings of 65 patients with SPG7 and 50 HCs from the LBW condition and of 57 patients and 37 HCs from the SFW condition were analyzed. Individual participant characteristics are provided in eTable 2. Most of the patients (61 of 65) exhibited clinical signs of both cerebellar and pyramidal system involvement (eFigure 2).

### Measures of Spatiotemporal Gait Variability Discriminate Between Patients and Controls With Large Effect Sizes Even in Patients With Mild Disease Severity

**Figure 1:**
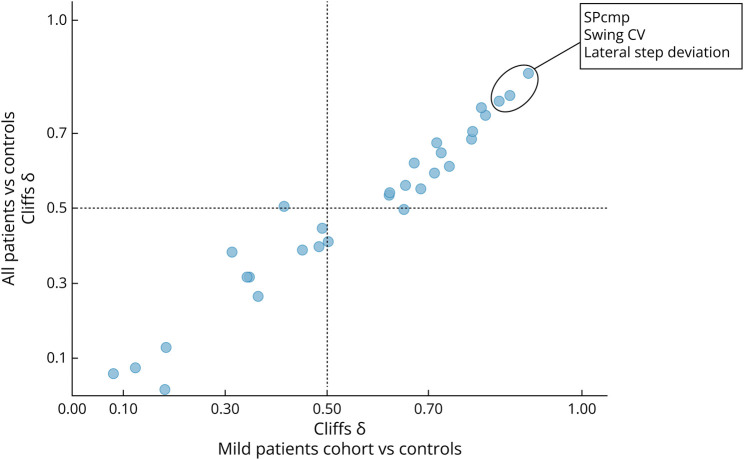
Discriminatory Power of 30 Gait Measures in Laboratory-Based Walking Scatter plot displaying discriminative effect size Cliff δ for all patients with SPG7 (y-axis) and the mild patient cohort (SPRS^mobility^ ≤ 9, x-axis) against healthy controls for each gait measure. Top 3 measures are highlighted. SPG7 = spastic paraplegia type 7; SPRS = Spastic Paraplegia Rating Scale; SPRS^mobility^ = mobility subscore of the SPRS (items 1–6)

In LBW, the comparison between patients with SPG7 and HCs yielded large discriminative effect sizes of |Cliff δ| ≥ 0.8 for 5 of 30 gait measures and at least moderate effect sizes of |δ| ≥ 0.5 for 18 of 30 gait measures. The strongest discrimination was observed for measures of spatial and temporal gait variability: SPcmp (δ = 0.90), swing CV (δ = 0.86), and lateral step deviation (δ = 0.84) (Figure 1, Table 1). Other measures displaying high discriminatory power included foot angle measures (pitch at initial contact: δ = 0.81; pitch at toe off: δ = 0.78) and measures of gait smoothness (HR V: δ = −0.80; HR AP: δ = −0.79).

**Table 1:** Discrimination Between Patients With SPG7 and HCs (Laboratory-Based Walking)

	SPG7	HC	
N (f/m)	65 (20/45)	50 (27/23)	
Demographic/clinical measures	Median (min–max)	Median (min–max)	*p* Value
Age (y)	53 (14–72)	47.5 (22–77)	0.15
Disease duration (y)	15 (1–57)		
SPRS^mobility^	9.0 (3–15)	0.0 (0–4)	
SPRS	14.0 (3–28)	0.0 (0–5)	
SARA^PG^	4.0 (1–7)	0.0 (0–1)	
SARA	9.0 (3.5–18)	0.0 (0–4)	
FARS-ADL	9.0 (2–19)	0.0 (0–2.5)	

Gait measures	Median	MADN	Median	MADN	Cliff δ	AUC	*p* Value
SPcmp	0.361	0.146	0.158	0.066	0.90	0.95	2.3e-16^a^
Swing CV	0.0236	0.0109	0.0121	0.0034	0.86	0.93	3.6e-15^a^
Lateral step deviation (%)	3.48	1.18	1.94	0.52	0.84	0.92	1.6e-14^a^
Pitch at initial contact (°)	18.7	5.7	27.5	4.8	−0.81	0.91	7.4e-13^a^
Harmonic ratio V	2.02	0.52	3.63	0.84	−0.80	0.90	1.8e-13^a^
Harmonic ratio AP	1.90	0.48	3.19	0.91	−0.79	0.89	5.9e-13^a^
Pitch at toe off (°)	29.5	6.8	37.9	4.0	−0.78	0.89	7e-13^a^
Stride time CV	0.0329	0.0176	0.0169	0.0067	0.74	0.87	1.2e-11^a^
Circumduction	5.05	1.83	2.79	0.95	0.72	0.86	3.3e-11^a^
Double support MADN	1.48	0.53	0.887	0.219	0.72	0.86	5.6e-11^a^
Stride length (m)	1.05	0.20	1.33	0.19	−0.71	0.86	7.3e-11^a^
Gait speed (m/s)	0.924	0.219	1.24	0.20	−0.68	0.84	3.6e-10^a^
Harmonic ratio ML	1.66	0.56	2.47	0.64	−0.67	0.84	7.6e-10^a^
Pitch at toe off MADN	1.35	0.48	0.791	0.295	0.65	0.83	2.1e-09^a^
Stride length CV	0.0423	0.0182	0.0255	0.0074	0.65	0.83	2.4e-09^a^
Double support (%)	25.4	4.7	19.5	2.7	0.62	0.81	1.2e-08^a^
Swing (%)	37.4	2.4	40.3	1.4	−0.62	0.81	1.2e-08^a^
Pitch at initial contact MADN	1.55	0.57	1.05	0.36	0.50	0.75	9e-06^a^
LRoM transverse (°)	12.7	5.4	8.94	2.70	0.49	0.75	6.8e-06^a^
Toe out angle MADN	2.11	0.90	1.47	0.66	0.48	0.74	9.4e-06^a^
Elevation at midswing MADN	0.358	0.145	0.235	0.067	0.45	0.73	3.5e-05^a^
Pitch at mid swing (°)	11.3	4.6	15.1	3.8	−0.42	0.71	0.00014^a^
Stride time (s)	1.15	0.11	1.07	0.06	0.36	0.68	0.00083^a^
LRoM sagittal (°)	6.58	2.36	4.78	1.42	0.35	0.67	0.0014^a^
LRoM sagittal CV	0.180	0.063	0.138	0.042	0.34	0.67	0.0017^a^
Elevation at midswing (cm)	1.57	0.73	1.18	0.48	0.31	0.66	0.0041
Toe out angle (°)	9.86	5.18	8.02	5.53	0.18	0.59	0.091
LRoM coronal CV	0.0952	0.0508	0.0896	0.0401	0.18	0.59	0.097
LRoM coronal (°)	7.20	2.12	7.69	2.18	−0.12	0.56	0.26
LRoM transverse CV	0.192	0.071	0.184	0.071	0.08	0.54	0.46

Abbreviations: CV = coefficient of variation; FARS-ADL = activities of daily living subscore of the Friedreich Ataxia Rating Scale; HC = healthy control; MADN = normalized median absolute deviation; SARA = Scale for the Assessment and Rating of Ataxia; SARA^PG^ = posture and gait subscore of SARA (items 1–4); SPG7 = spastic paraplegia type 7; SPRS = Spastic Paraplegia Rating Scale; SPRS^mobility^ = mobility subscore of the SPRS (items 1–6).

a *p* < 0.05/n = 30: number of gait measures (Bonferroni).

To evaluate the ability of the candidate measures to discriminate patients with mild disease severity from HCs, we performed a median split of the patient cohort with respect to the SPRS^mobility^ scale. For this mild patient cohort (SPRS^mobility^ ≤ 9, n = 41), discrimination with large effect sizes was observed for 2 of 30 measures, and at least moderate effect sizes for 17 of 30 measures (eTable 3). The strongest discrimination was observed for the measures SPcmp (δ = 0.86), swing CV (δ = 0.80), and lateral step deviation (δ = 0.78), mirroring the results for the whole patient cohort with minor decreases in effect size (Figure 1). Comparing the sets of measures with at least moderate effect size between the whole patient cohort and the mild patient cohort, a large overlap was apparent (16 measures identical). We did not identify a measure that exhibited large discriminative power only in the mild patient cohort.

### Measures of Spatiotemporal Gait Variability Correlate With Clinical Measures of Mobility Even in Patients With Mild Disease Severity

**Figure 2:**
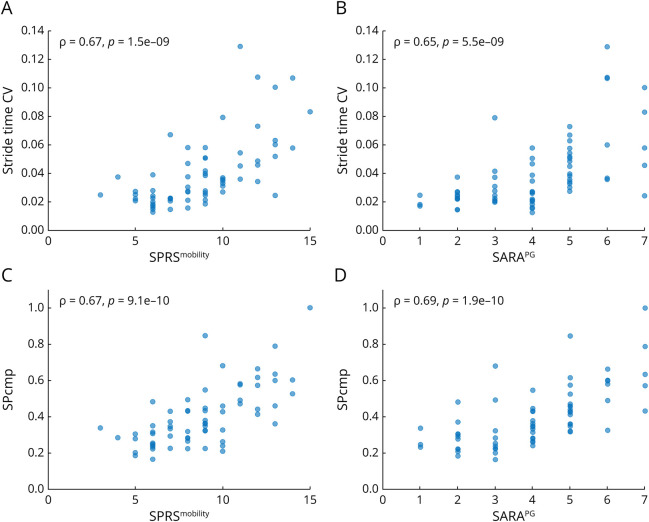
Top Correlations Between Gait Measures and Clinical Measures in All Patients With SPG7 During Laboratory-Based Walking (A, B) Stride time CV and (C, D) SPcmp vs SPRS^mobility^ (left) and SARA^PG^ (right). The Spearman ρ and associated *p* value are depicted in the upper left corner. CV = coefficient of variation; SARA = Scale for the Assessment and Rating of Ataxia; SARA^PG^ = posture and gait subscore of SARA (items 1–4); SPG7 = spastic paraplegia type 7; SPRS = Spastic Paraplegia Rating Scale; SPRS^mobility^ = mobility subscore of the SPRS (items 1–6).

For the at least moderately discriminative gait measures in the whole patient cohort (18 measures) and mild patient cohort (17 measures), we assessed correlations with clinician-reported measures (primary outcomes: SPRS^mobility^, SARA^PG^; exploratory outcomes: SPRS, SARA, FARS-ADL). Discriminative gait measures correlated with clinician-reported measures of mobility and posture and gait, with large effect sizes. For the mobility scale SPRS^mobility^, the largest effect sizes were observed for correlations with measures of spatiotemporal gait variability such as the spatial variability composite measure SPcmp (ρ = 0.67, *p* = 9.1e-10), stride time CV (ρ = 0.67, *p* = 1.5e-9), and swing CV (ρ = 0.64, *p* = 1.1e-8) (Figure 2, Table 2). The same gait measures also correlated with SARA^PG^, with only minor differences of effect sizes. For the gait measures stride length, gait speed, pitch at toe off, double support, and swing, however, significantly larger effect sizes were observed for the correlations with SPRS^mobility^ than with SARA^PG^. On the contrary, the measure of foot angle variability pitch at toe off MADN correlated more strongly with SARA^PG^ than with SPRS^mobility^.

**Table 2:** Correlation of Discriminative Gait Measures With Primary Clinical Measures (Laboratory-Based Walking; All Patients [Left] and Mild Patient Cohort [Right])

	All patients (N = 65)		Mild patient cohort (N = 41)	
	SPRS^mobility^	SARA^PG^	SPRS^mobility^	SARA^PG^
SPcmp	0.67 (0.50 to 0.79)^a^	0.69 (0.52 to 0.81)^a^	0.50 (0.22 to 0.69)^a^	0.48 (0.21 to 0.67)^a^
Stride time CV	0.67 (0.51 to 0.78)^a^	0.65 (0.47 to 0.77)^a^	0.38 (0.08 to 0.59)	0.46 (0.16 to 0.67)
Swing CV	0.64 (0.46 to 0.76)^a^	0.60 (0.40 to 0.74)^a^	0.53 (0.24 to 0.71)^a^	0.35 (0.01 to 0.61)
Stride length CV	0.64 (0.45 to 0.76)^a^	0.60 (0.38 to 0.73)^a^	—	—
Stride length (m)	−0.63 (−0.76 to −0.42)^a^	−0.39 (−0.60 to −0.13)^a^	−0.34 (−0.60 to −0.03)	−0.14 (−0.44 to 0.20)
Gait speed (m/s)	−0.61 (−0.75 to −0.41)^a^	−0.39 (−0.58 to −0.13)^a^	−0.29 (−0.58 to 0.06)	−0.15 (−0.44 to 0.22)
Pitch at toe off (°)	−0.57 (−0.70 to −0.40)^a^	−0.46 (−0.65 to −0.22)^a^	−0.35 (−0.58 to −0.03)	−0.36 (−0.62 to −0.04)
Lateral step deviation (%)	0.51 (0.29 to 0.68)^a^	0.52 (0.28 to 0.69)^a^	0.22 (−0.12 to 0.49)	0.21 (−0.14 to 0.50)
Double support (%)	0.48 (0.27 to 0.65)^a^	0.33 (0.07 to 0.53)	0.26 (−0.03 to 0.51)	0.24 (−0.08 to 0.52)
Swing (%)	−0.46 (−0.63 to −0.23)^a^	−0.32 (−0.53 to −0.05)	−0.27 (−0.53 to 0.00)	−0.25 (−0.53 to 0.09)
Double support MADN	0.42 (0.18 to 0.61)^a^	0.44 (0.22 to 0.62)^a^	0.38 (0.06 to 0.60)	0.28 (−0.05 to 0.55)
Pitch at initial contact (°)	−0.39 (−0.60 to −0.16)	−0.28 (−0.50 to −0.03)	−0.11 (−0.41 to 0.24)	0.05 (−0.26 to 0.35)
Pitch at toe off MADN	0.37 (0.16 to 0.56)	0.49 (0.33 to 0.65)^a^	0.44 (0.17 to 0.63)	0.43 (0.08 to 0.66)
Pitch at initial contact MADN	0.35 (0.11 to 0.56)	0.36 (0.10 to 0.58)	—	—
Harmonic ratio AP	−0.34 (−0.53 to −0.09)	−0.33 (−0.55 to −0.07)	−0.06 (−0.38 to 0.26)	−0.07 (−0.37 to 0.26)
Circumduction	0.33 (0.13 to 0.51)	0.16 (−0.12 to 0.40)	0.11 (−0.19 to 0.41)	−0.12 (−0.41 to 0.21)
Harmonic ratio V	−0.27 (−0.50 to −0.02)	−0.23 (−0.47 to 0.05)	−0.06 (−0.37 to 0.27)	0.08 (−0.20 to 0.36)
Harmonic ratio ML	−0.22 (−0.43 to 0.01)	−0.14 (−0.39 to 0.11)	−0.12 (−0.40 to 0.19)	−0.03 (−0.29 to 0.30)
Pitch at mid swing (°)	—	—	−0.18 (−0.45 to 0.16)	−0.09 (−0.38 to 0.20)

Abbreviations: CV = coefficient of variation; SPRS^mobility^ = mobility subscore of the SPRS (items 1–6); SARA^PG^ = posture and gait subscore of SARA (items 1–4).

Spearman ρ (95% CI).

a *p* < 0.05/(2 × 18 [all patients] or 17 [mild patient cohort]).

Even within the mild patient cohort, correlations between gait measures and clinician-reported measures of mobility, posture, and gait were observed with medium-to-large effect sizes. Specifically, 3 gait measures correlated with SPRS^mobility^—swing CV (ρ = 0.53, *p* = 4.2e-4), SPcmp (ρ = 0.50, *p* = 9.5e-4), and stride length CV (ρ = 0.48, *p* = 0.0014) (visualization in eFigure 3)—and 1 gait measure correlated with SARA^PG^—SPcmp (ρ = 0.48, *p* = 0.0014) (Table 2). Of note, within the mild patient cohort, all gait measures with significant correlations belonged to the domain of spatiotemporal gait variability.

For standard clinical measures of HSP-related and ataxia-related disease severity, correlations with large effect sizes of ρ ≥ 0.5 were primarily observed for measures of spatiotemporal gait variability (eTable 4). Specifically, the correlations of the largest effect sizes with HSP-related disease severity (SPRS) were observed for SPcmp, stride time CV, and stride length CV. The measures stride length CV, stride time CV, and swing CV displayed the largest effect sizes in the correlation with ataxia-related disease severity (SARA). Several of the measures correlated with a measure of patient-relevant impairment in everyday life activities (FARS-ADL), with stride length CV (ρ = 0.45, *p* = 1.9e-4), swing CV (ρ = 0.44, *p* = 2.7e-4), and gait speed (ρ = −0.41, *p* = 7.6e-4) leading the list. In addition, the previously highlighted measures stride time CV (ρ = 0.38, *p* = 0.0019) and SPcmp (ρ = 0.39, *p* = 0.0015) correlated with FARS-ADL (eFigure 4). Correlations of gait measures with SPRS, SARA, and FARS-ADL within the mild patient cohort are provided in eTable 5.

**Figure 3:**
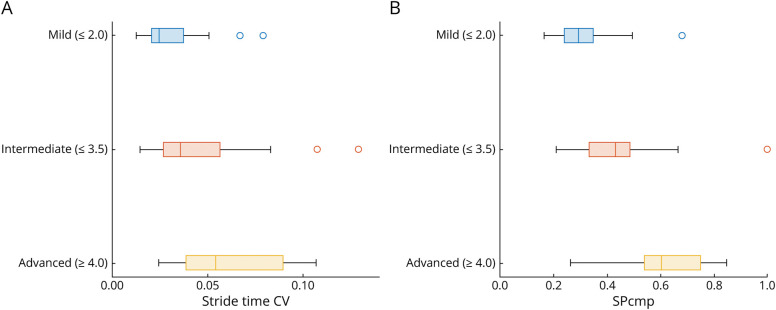
Box Plots With Values of (A) Stride Time CV and (B) SPcmp in Groups of Patients in Different Disease Stages, Defined by FARS Staging (Mild: ≤2; Intermediate: 2.5–3.5; Advanced: ≥4) Line inside boxes = median, edges of boxes = lower and upper quartiles, whiskers = (nonoutlier) minimum and maximum, circles = outliers (values >1.5 × interquartile range away from lower/upper edge of the box). CV = coefficient of variation; FARS = Friedreich Ataxia Rating Scale.

Comparison of groups of patients in different disease stages defined by the FARS staging (mild: ≤2.0, intermediate: 2.5–3.5, advanced: ≥4.0) revealed significant group differences between mild, intermediate, and advanced stages for SPcmp (*p*_Kruskal-Wallis_ = 1.1e-4; mild vs intermediate: *p* = 7.4e-4, δ = 0.52, AUC = 0.76; intermediate vs advanced: *p* = 0.022, δ = 0.57, AUC = 0.78) and between mild and intermediate stages for stride time CV (*p*_Kruskal-Wallis_ = 2.2e-4, mild vs intermediate: *p* = 8.6e-5, δ = 0.60, AUC = 0.80; intermediate vs advanced: *p* = 0.71, δ = 0.10, AUC = 0.55). Figure 3 illustrates the thus defined approximate meaningful score regions of the 2 gait measures.

### Measures of Pace and Gait Variability Discriminate Patients From Controls and Correlate With Clinician-Reported Measures of Mobility Under Conditions Simulating Real Life

To assess performance of gait measures in simulated real life, discrimination of patients from controls and correlation with clinician-reported outcomes were assessed for data recorded under the SFW condition. Gait measures discriminated patients from HCs in SFW with large effect sizes for 3 of 30 measures and with at least moderate effect sizes for 17 of 30 measures (Table 3). The most discriminative measures—HR V (δ = −0.85), HR AP (δ = −0.80), swing CV (δ = 0.83)—capture gait smoothness and temporal variability. In contrast to LBW, measures of spatial gait variability (lateral step deviation, stride length CV, SPcmp) were not among the top measures but still discriminated with moderate effect sizes.

**Table 3:** Discrimination of Patients With SPG7 From HCs (Supervised Free Walking)

	SPG7	HC	
N (f/m)	57 (16/41)	37 (22/15)	
Demographic/clinical measures	Median (min–max)	Median (min–max)	*p* Value
Age (y)	50 (14–72)	46 (22–77)	0.56
Disease duration (y)	13 (2–57)		
SPRS^mobility^	8.0 (3–15)	0.0 (0–4)	
SPRS	14.0 (3–26)	0.0 (0–5)	
SARA^PG^	4.0 (1–7)	0.0 (0–1)	
SARA	8.5 (3.5–18)	0.0 (0–4)	
FARS-ADL	9.0 (2–19)	0.0 (0–2.5)	

Gait measures	Median	MADN	Median	MADN	Cliff δ	AUC	*p* Value
SPcmp	0.434	0.119	0.281	0.112	0.58	0.79	2.1e-06^a^
Swing CV	0.0237	0.0081	0.0155	0.0034	0.83	0.91	1.7e-11^a^
Lateral step deviation (%)	4.09	0.86	3.07	0.68	0.57	0.78	3.8e-06^a^
Pitch at initial contact (°)	18.0	7.9	26.5	4.9	−0.74	0.87	1.5e-09^a^
Harmonic ratio V	1.95	0.49	3.39	0.77	−0.85	0.93	3.1e-12^a^
Harmonic ratio AP	1.93	0.48	3.10	0.85	−0.80	0.90	5.9e-11^a^
Pitch at toe off (°)	32.7	5.8	39.1	4.1	−0.73	0.86	3.1e-09^a^
Stride time CV	0.0351	0.0143	0.0221	0.0083	0.62	0.81	4.7e-07^a^
Circumduction	4.98	2.07	2.69	0.84	0.78	0.89	1.7e-10^a^
Double support MADN	1.65	0.54	1.09	0.30	0.75	0.87	1.2e-09^a^
Stride length (m)	1.11	0.22	1.35	0.20	−0.64	0.82	1.5e-07^a^
Gait speed (m/s)	0.994	0.223	1.25	0.23	−0.65	0.82	1.4e-07^a^
Harmonic ratio ML	1.77	0.44	2.44	0.37	−0.71	0.86	5.6e-09^a^
Pitch at toe off MADN	1.51	0.61	0.925	0.355	0.67	0.84	4.1e-08^a^
Stride length CV	0.0468	0.0172	0.0309	0.0098	0.62	0.81	5.1e-07^a^
Double support (%)	25.3	4.6	20.3	2.8	0.59	0.79	1.8e-06^a^
Swing (%)	37.4	2.1	39.9	1.4	−0.59	0.79	1.7e-06^a^
Pitch at initial contact MADN	1.72	0.70	1.48	0.58	0.41	0.70	0.00093^a^
LRoM transverse (°)	11.7	4.4	10.1	4.1	0.29	0.64	0.018
Toe out angle MADN	2.32	0.69	1.91	0.51	0.47	0.74	0.00012^a^
Elevation at midswing MADN	0.446	0.146	0.322	0.116	0.48	0.74	9.6e-05^a^
Pitch at mid swing (°)	12.1	3.4	15.0	4.0	−0.32	0.66	0.01
Stride time (s)	1.11	0.09	1.07	0.09	0.27	0.63	0.03
LRoM sagittal (°)	6.38	2.28	5.09	1.68	0.43	0.72	0.00046^a^
LRoM sagittal CV	0.192	0.055	0.196	0.077	0.05	0.53	0.67
Elevation at midswing (cm)	1.61	0.82	1.12	0.53	0.41	0.70	0.00093^a^
Toe out angle (°)	11.6	4.3	10.1	7.6	0.15	0.58	0.22
LRoM coronal CV	0.114	0.056	0.109	0.029	0.08	0.54	0.51
LRoM coronal (°)	7.56	2.18	7.36	3.00	−0.02	0.51	0.88
LRoM transverse CV	0.234	0.073	0.286	0.094	−0.22	0.61	0.076

Abbreviations: CV = coefficient of variation; FARS-ADL = activities of daily living subscore of the Friedreich Ataxia Rating Scale; HC = healthy control; MADN = normalized median absolute deviation; SARA = Scale for the Assessment and Rating of Ataxia; SARA^PG^ = posture and gait subscore of SARA (items 1–4); SPG7 = spastic paraplegia type 7; SPRS = Spastic Paraplegia Rating Scale; SPRS^mobility^ = mobility subscore of the SPRS (items 1–6).

a *p* < 0.05/n = 30: number of gait measures (Bonferroni).

At least large correlations between the LBW and SFW conditions were observed for 27 of 30 gait measures (very large: 17, large: 10, medium: 3; eTable 6). Consequently, the same set of measures largely discriminated patients from controls in SFW as in LBW (although the rankings within the set of discriminative measures differed between the 2 conditions). Specifically, except pitch at initial contact MADN, all measures discriminative with at least moderate effect size in LBW were reproduced in SFW. Reversely, all measures with at least moderate effect sizes in SFW were also found to be discriminative in LBW. For 3 representative participants, values of 5 key gait measures in LBW and SFW are presented in eFigure 5.

**Figure 4:**
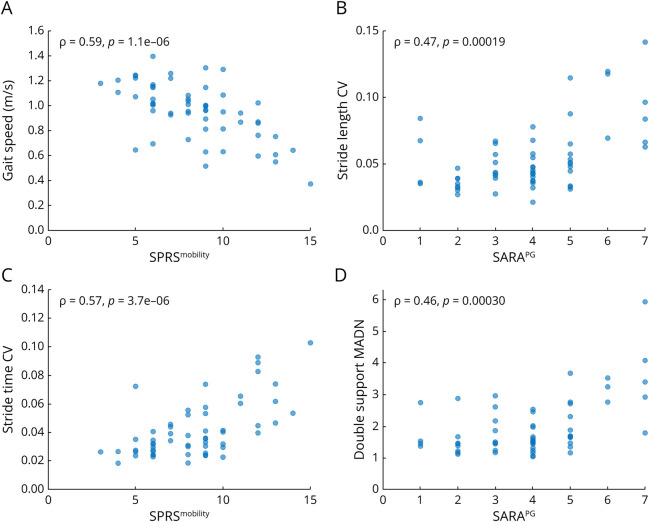
Top Correlations Between Gait Measures and Clinical Measures in All Patients With SPG7 During Supervised Free Walking (A) Gait speed and (C) stride time CV vs SPRS^mobility^; (B) stride length CV and (D) double support MADN vs SARA^PG^. The Spearman ρ and associated *p* value are depicted in the upper right/left corner. CV = coefficient of variation; MADN = normalized median absolute deviation; SARA = Scale for the Assessment and Rating of Ataxia; SARA^PG^ = posture and gait subscore of SARA (items 1–4); SPG7 = spastic paraplegia type 7; SPRS = Spastic Paraplegia Rating Scale; SPRS^mobility^ = mobility subscore of the SPRS (items 1–6).

Gait measures discriminative in SFW correlated with the mobility measure SPRS^mobility^ with large effect sizes. In contrast to the results for LBW, 2 of the 3 top measures—gait speed (ρ = −0.59, *p* = 1.1e-6), stride time CV (ρ = 0.57, *p* = 3.7e-06), and stride length (ρ = −0.55, *p* = 1.2e-05)—were measures of pace rather than gait variability (Figure 4, eTable 7). Still, all measures of spatiotemporal gait variability correlated with SPRS^mobility^, except for lateral step deviation. Correlations with SARA^PG^ were found for the measures stride length CV (ρ = 0.47, *p* = 1.9e-04), double support MADN (ρ = 0.46, *p* = 3.0e-4), and pitch at toe off MADN (ρ = 0.46, *p* = 3.2e-4).

Top measures in the correlation with HSP-related disease severity (SPRS) were stride time CV, gait speed, and pitch at initial contact, all displaying moderate effect sizes (eTable 7). Correlations with ataxia-related disease severity (SARA) were found for double support MADN, pitch at toe off MADN (both with large effect sizes), and stride length CV. Correlations with FARS-ADL were found for measures of pace and temporal and foot angle variability, with moderate effect sizes for gait speed and double support.

## Discussion

In this cross-sectional, multicenter study, we used body-worn sensors to identify candidate digital gait outcomes for upcoming treatment trials for SPG7. The study's design, encompassing data capture across multiple sites, and gait assessments in both laboratory and simulated real-life conditions closely mirrored anticipated settings in future clinical trials. We established a shortlist of promising gait measures through a structured multistep approach. Beginning with a hypothesis-based preselection of gait measures informed by the literature and clinical experience, we subsequently narrowed down the list to measures with high discriminative power, finally selecting those that exhibited the strongest correlation with clinical measures of patient-relevant health aspects.

The gait measures that demonstrated the largest correlations with the SPRS^mobility^ and SARA^PG^ were related to spatiotemporal gait variability, as exemplified by the spatial variability composite measure SPcmp (SPRS^mobility^: Spearman ρ = 0.67; SARA^PG^: ρ = 0.69) and stride time CV (SPRS^mobility^: ρ = 0.67; SARA^PG^: ρ = 0.65). At the same time, these parameters also discriminated between patients and controls (SPcmp: Cliff δ = 0.90; stride time CV: δ = 0.74). These results corroborate and extend findings from previous studies performed in mixed or genotype-specific cohorts of patients with cerebellar ataxias. In a previous study with a mixed cohort of patients with cerebellar ataxias, gait measures such as variability of stride length and of stride duration have demonstrated discriminative power between patients and controls and have shown correlations with the SARA^PG^.^3^ In another study with a mixed cohort of patients with SCA, spatial and temporal stride variability measures discriminated between patients and controls.^6^ In the same study, variability of the double support phase was among the measures that correlated most strongly with the SARA in patients with SCA. The other top measures were, however, related to foot angles and their variability, measures that were not among the prime candidates in this SPG7 study.

Our results, highlighting measures of spatiotemporal variability as prime candidate gait outcomes, also extend the findings of the very few published studies using sensor-based gait analysis in HSP. In the largest study, measures of temporal stride variability such as stride time CV discriminated patients from controls and demonstrated cross-sectional correlation with SPRS.^9^ However, they did not show longitudinal progression on follow-up assessments.^8^ The cited studies were limited, however, by the inclusion of a genotypically mixed and thus phenotypically heterogeneous cohort of patients with HSP. The cohort included 17 patients with SPG7, but no genotype-specific subgroup analysis was presented. The study was further limited by analysis of only temporal and gait cycle measures, and not spatial or foot angle measures. Moreover, it included patients dependent on walking aids—also during the measurements, which may have a profound impact on gait measures—and was thus of reduced informative value for trial-relevant mild disease stages.

Taken together, our results extend the utility of spatiotemporal gait variability measures to capture disease-related gait impairment from cerebellar ataxias—and from HSP, although here only partially and much less established—to the spastic ataxia SPG7. A priori, this transferability was not self-evident because it was unclear how the variable spastic component of gait in SPG7 adds to or modifies gait features one would observe in pure ataxic gait and vice versa. This finding may extend to other spastic ataxias, making the gait measures identified in this study potential candidate outcomes for other genotypes as well.

Several gait measures evaluated in this study discriminated between patients with SPG7 with mild disease severity and controls and demonstrated correlations with clinician-reported outcome assessments even *within* this mild cohort. This is of particular importance because future disease-modifying treatment trials will likely focus on patients in mild disease stages. Widely used clinical outcome scales in ataxia and HSP—SARA and SPRS—have been designed to capture the full disease spectrum. Therefore, they may have limited sensitivity to change and are prone to floor effects in these early stages. Digital gait measures on the contrary may allow monitoring of disease progression within trial-relevant time frames of 1–2 years even in patients with mild disease.^4^ The gait measures identified in this study, demonstrating strong discriminative power and correlation with clinical scales in mild disease stages, are thus prime candidate outcomes for potential treatment trials—complementing clinical scale assessments and other outcome modalities.

To serve as meaningful outcomes in treatment trials, digital-motor measures must not merely capture change over time but also reflect changes relevant to patients. Mobility is a concept that is known to be of particularly high relevance for patients with HSP^27^ and ataxia.^28-30^ The robust correlations with the SPRS^mobility^ scale, therefore, indicate that our top gait measures indeed mirror disease aspects that are meaningful to patients. However, the extent to which gait assessments in controlled laboratory conditions accurately capture aspects of mobility that are relevant to patients in real life was unknown. To address this limitation, this study included assessments of “supervised free walking” conducted outside the laboratory but within institutional compounds and under supervision by staff members. This walking condition thus simulates more complex real-life conditions and allows for more variability, potentially reducing effect sizes while concurrently maintaining stringent control to ensure technical robustness. Remarkably, gait measures discriminated patients and controls and correlated with clinical measures in the SFW condition although the walking routes naturally differed between the multiple participating centers. In comparison with LBW, only minor reductions of effect sizes were observed in SFW that were greater for variability measures than for measures of pace, which is likely explained by the inherently larger variability during SFW. The successful application of this assessment approach in SPG7 opens up 2 distinct perspectives: First, by showing that relevant gait measures could be captured in more variable conditions simulating real life, this study paves the way toward real-life measurements in patients' everyday lives, which in turn would offer a maximum of ecological validity. Second, assessments of “supervised free walking” could serve as a means to increase ecological validity of digital gait outcomes in multicenter treatment trials while simultaneously avoiding the technical challenges associated with real-life measurements.

The 2 top gait measures, stride time CV and SPcmp, differentiated between groups of patients defined by a staging of disease severity on a functional, patient-relevant level (FARS staging). The ranges of values associated with each disease stage could thus be interpreted as approximate meaningful score regions, a concept that has recently been endorsed by the FDA.^10^ Consequently, establishing the association between ranges of values of gait measures and disease stages defined through patient-relevant health aspects underlines the patient meaningfulness of these gait measures.

This study has several limitations. The patient cohort consisted of significantly more men than women while the sex ratio in the control cohort was more balanced. While we did not find a significant influence of sex on the gait measures analyzed in this study (neither in HCs, nor in patients; analysis not included in this article), the results of this study may be less representative for women than for men. Moreover, while good-to-excellent test-retest reliability has already been shown in ataxia populations in earlier studies for the most sensitive and specific gait measures also highlighted in this study,^6,31^ future studies are warranted to demonstrate test-retest reliability also specifically for SPG7. Owing to its cross-sectional design, it could not evaluate responsiveness to change, a crucial criterion for the viability of gait measures as outcomes in treatment trials. Consequently, the measures identified here will need to be evaluated longitudinally. Furthermore, while the results of this study indicate that the identified gait measures could reflect health aspects that are meaningful to patients, more work is needed to establish the meaningfulness of these gait measures.

In conclusion, this study identified multicenter applicable digital gait measures capable of discriminating patients and controls and correlating with relevant COAs, even in mild disease stages, and in settings simulating real life. If validated longitudinally, the measures are prime candidate outcomes for future treatment trials for SPG7 and other spastic ataxias.

## Appendix 1 Authors

**Table:**

Name	Location	Contribution
Lukas Beichert, MD	Division Translational Genomics of Neurodegenerative Diseases, Hertie-Institute for Clinical Brain Research and Center for Neurology, and German Center for Neurodegenerative Diseases (DZNE), University of Tübingen, Germany	Drafting/revision of the manuscript for content, including medical writing for content; analysis or interpretation of data
Jens Seemann, MSc	Section Computational Sensomotorics, Hertie Institute for Clinical Brain Research; Centre for Integrative Neuroscience (CIN), Tübingen, Germany	Drafting/revision of the manuscript for content, including medical writing for content; analysis or interpretation of data
Christoph Kessler, MD	Department of Neurodegenerative Diseases, Hertie-Institute for Clinical Brain Research and Center for Neurology, University of Tübingen, Germany	Drafting/revision of the manuscript for content, including medical writing for content; major role in the acquisition of data
Andreas Traschütz, MD, PhD	Division Translational Genomics of Neurodegenerative Diseases, Hertie-Institute for Clinical Brain Research and Center for Neurology, and German Center for Neurodegenerative Diseases (DZNE), University of Tübingen, Germany	Drafting/revision of the manuscript for content, including medical writing for content; major role in the acquisition of data
Doreen Müller	Division Translational Genomics of Neurodegenerative Diseases, Hertie-Institute for Clinical Brain Research and Center for Neurology, and German Center for Neurodegenerative Diseases (DZNE), University of Tübingen, Germany	Major role in the acquisition of data
Katrin Dillmann-Jehn	German Center for Neurodegenerative Diseases (DZNE), University of Tübingen; Center for Neurology and Hertie Institute for Clinical Brain Research, University Hospital Tübingen, Germany	Major role in the acquisition of data
Ivana Ricca, MD	Molecular Medicine, IRCCS Fondazione Stella Maris, Pisa, Italy	Drafting/revision of the manuscript for content, including medical writing for content; major role in the acquisition of data
Sara Satolli, MD	Molecular Medicine, IRCCS Fondazione Stella Maris, Pisa, Italy	Drafting/revision of the manuscript for content, including medical writing for content; major role in the acquisition of data
Nazli A. Basak, PhD	Koç University, Translational Medicine Research Center, KUTTAM-NDAL, Istanbul, Turkey	Drafting/revision of the manuscript for content, including medical writing for content; major role in the acquisition of data
Giulia Coarelli, MD, PhD	Sorbonne Université, Paris Brain Institute, INSERM, CNRS, APHP, Paris, France	Drafting/revision of the manuscript for content, including medical writing for content; major role in the acquisition of data
Dagmar Timmann, MD	Department of Neurology and Center for Translational Neuro- and Behavioral Sciences, University Hospital Essen, University of Duisburg-Essen, Germany	Drafting/revision of the manuscript for content, including medical writing for content; major role in the acquisition of data
Cynthia Gagnon, PhD	Groupe de recherche interdisciplinaire sur les maladies neuromusculaires (GRIMN), Centre intégré universitaire de santé et de services sociaux du Saguenay-Lac-St-Jean; Centre de recherche du Centre intégré universitaire de santé et de services sociaux du Saguenay-Lac-St-Jean; Faculté de médecine et des sciences de la santé, Université de Sherbrooke, Québec, Canada	Drafting/revision of the manuscript for content, including medical writing for content; major role in the acquisition of data
Bart P.C. van de Warrenburg, MD, PhD	Department of Neurology, Radboud University Medical Center, Nijmegen, the Netherlands	Drafting/revision of the manuscript for content, including medical writing for content; major role in the acquisition of data
Winfried Ilg, PhD	Section Computational Sensomotorics, Hertie Institute for Clinical Brain Research; Centre for Integrative Neuroscience (CIN), Tübingen, Germany	Drafting/revision of the manuscript for content, including medical writing for content; analysis or interpretation of data
Matthis Synofzik, MD	Division Translational Genomics of Neurodegenerative Diseases, Hertie-Institute for Clinical Brain Research and Center for Neurology, and German Center for Neurodegenerative Diseases (DZNE), University of Tübingen, Germany	Drafting/revision of the manuscript for content, including medical writing for content; major role in the acquisition of data; study concept or design; analysis or interpretation of data
Rebecca Schüle, MD	German Center for Neurodegenerative Diseases (DZNE), University of Tübingen; Center for Neurology and Hertie Institute for Clinical Brain Research, University Hospital Tübingen; Division of Neurodegenerative Diseases, Department of Neurology, Heidelberg University Hospital, Germany	Drafting/revision of the manuscript for content, including medical writing for content; major role in the acquisition of data; study concept or design; analysis or interpretation of data

## Appendix 2 Coinvestigators of the PROSPAX Consortium

**Table:**

Name	Location	Role	Contribution
Filippo M. Santorelli, MD	Molecular Medicine, IRCCS Fondazione Stella Maris, Pisa, Italy	Principal Site Investigator	Site coordination, clinical assessments
Sirio Cocozza, MD	Federico II University Naples, Italy	Site Investigator	Site coordination
Atay Vural, MD, PhD	University, Department of Neurology, Istanbul, Turkey	Site Investigator	Clinical assessments
Özgür Öztop Çakmak, MD	Koç University, Department of Neurology and Center for, Istanbul, Turkey	Site Investigator	Clinical assessments
Nazan Akkaya, MD	Koç University, Translational Medicine Research Center (KUTTAM), Istanbul, Turkey	Site Investigator	Clinical assessments
Kardelen Akar	Koç University, Translational Medicine Research Center (KUTTAM), Istanbul, Turkey	Site Investigator	Clinical assessments, digital-motor assessments
Sophie Tezenas du Montcel	Sorbonne Université, Paris Brain Institute, INSERM, CNRS, APHP, Paris, France	Principal Site Investigator	Site coordination
Alexandra Durr	Sorbonne Université, Paris Brain Institute, INSERM, CNRS, APHP, Paris, France	Site Investigator	Site coordination, clinical assessments
Claire Ewenczyk	Sorbonne Université, Paris Brain Institute, INSERM, CNRS, APHP, Paris, France	Site Investigator	Clinical assessments
Anna Heinzmann	Sorbonne Université, Paris Brain Institute, INSERM, CNRS, APHP, Paris, France	Site Investigator	Clinical assessments
Lucie Pierron	Sorbonne Université, Paris Brain Institute, INSERM, CNRS, APHP, Paris, France	Site Investigator	Clinical assessments
Paulina Cunha	Sorbonne Université, Paris Brain Institute, INSERM, CNRS, APHP, Paris, France	Site Investigator	Clinical assessments
Hortense Hurmic	Sorbonne Université, Paris Brain Institute, INSERM, CNRS, APHP, Paris, France	Site Investigator	Clinical assessments
Stephan Klebe, MD	Department of Neurology and Center for Translational Neuro- and Behavioral Sciences, University Hospital Essen, University of Duisburg-Essen, Germany	Principal Site Investigator	Site coordination, clinical assessments

## Glossary

AP: anterior-posterior
AUC: area under the curve
CV: coefficient of variation
FARS-ADL: activities of daily living subscore of the Friedreich Ataxia Rating Scale
HCs: healthy control
HR: harmonic ratio
HSP: hereditary spastic paraplegia
LBW: laboratory-based walking
MADN: normalized median absolute deviation
ML: mediolateral
ROC: receiver operating characteristic
SCA1/SCA3: spinocerebellar ataxia types 1 and 3
SARA: Scale for the Assessment and Rating of Ataxia
SARA^PG^: posture and gait subscore of SARA (items 1–4)
SFW: supervised free walking
SPG7: spastic paraplegia type 7
SPRS: Spastic Paraplegia Rating Scale
SPRS^mobility^: mobility subscore of the SPRS (items 1–6)
V: vertical
